# Intragraft Hyaluronan Increases in Association With Acute Lung Transplant Rejection

**DOI:** 10.1097/TXD.0000000000001138

**Published:** 2021-03-22

**Authors:** Haley P. Hostetler, Megan L. Neely, Francine L. Kelly, John A. Belperio, Marie Budev, John M. Reynolds, Pali D. Shah, Lianne G. Singer, Laurie D. Snyder, Scott M. Palmer, Jamie L. Todd

**Affiliations:** 1 Division of Pulmonary, Allergy, and Critical Care Medicine, Duke University Medical Center, Durham, NC.; 2 Department of Biostatistics and Bioinformatics, Duke University Medical Center, Durham, NC.; 3 Duke Clinical Research Institute, Durham, NC.; 4 Department of Medicine, University of California Los Angeles, Los Angeles, CA.; 5 Department of Pulmonary Medicine, Cleveland Clinic, Cleveland, OH.; 6 Department of Medicine, Johns Hopkins University, Baltimore, MD.; 7 Department of Medicine, Toronto Lung Transplant Program, University Health Network, University of Toronto, Toronto, ON, Canada.

## Abstract

**Background.:**

Acute perivascular rejection (AR) is common in lung recipients and increases the risk for chronic lung allograft dysfunction (CLAD). Hyaluronan (HA), an extracellular matrix constituent, accumulates in experimental AR and can act as an innate immune agonist, breaking tolerance and potentiating alloimmunity. We previously demonstrated HA accumulates in CLAD after human-lung transplantation. We sought to determine if HA accumulates in the bronchoalveolar lavage fluid (BALF) concurrent with AR in lung recipients.

**Methods.:**

The cohort consisted of 126 first adult lung recipients at 5 transplant centers with a total of 373 BALF samples collected within the first posttransplant year. All samples were paired with a lung biopsy from the same bronchoscopy. BALF HA (ng/mL) was quantified by ELISA and log-transformed for analysis. Linear-mixed effect models, adjusted for potential confounders, were used to estimate the association between BALF HA concentration and the presence of AR on biopsy. The association between early posttransplant BALF HA levels and the development of CLAD was explored utilizing tertiles of maximum BALF HA level observed within the first 6 months of transplant.

**Results.:**

In analyses adjusted for potential confounders, BALF HA concentration was significantly increased in association with AR (change in means on log-scale 0.31; 95% CI, 0.01-0.60; *P* = 0.044). When considered on the original scale (ng/mL), BALF HA concentrations were 1.36 times (36%) higher, on average, among samples with, versus without, AR. The cumulative incidence of CLAD was numerically higher in individuals in the highest tertiles of BALF HA level within the first 6 months after transplant, as compared with those in the lowest tertile; however, this difference was not statistically significant (*P* = 0.32).

**Conclusions.:**

These results demonstrate accumulation of HA in clinical AR and suggest a mechanism by which innate and adaptive immune activation might interact in the development of AR and CLAD.

## Abstract 2

Supplemental Digital Content is available in the text.

## INTRODUCTION

Lung transplantation is an established treatment for select patients with end-stage lung disease; however, long-term survival remains limited with a median posttransplant life expectancy of about 6 years.^1^ Chronic lung allograft dysfunction (CLAD) represents the leading cause of late death after lung transplantation.^1^ Many studies suggest acute perivascular cellular rejection (AR) is a principal CLAD risk factor,^2,3^ and registry data indicate AR occurs frequently with approximately one-third of lung recipients experiencing at least 1 AR episode within the first posttransplant year.^1^ Given the frequency and implications of AR on long-term graft outcomes, understanding the mechanisms contributing to AR development despite maintenance immunosuppression is essential.

Hyaluronan (HA) is an extracellular matrix component that exists as a high molecular–weight polymer in homeostasis.^4-7^ In response to tissue damage, however, HA fragments are generated by the enzymatic action of hyaluronidases or synthesized de novo by local structural cells.^4,8^ Recent findings suggest such HA fragments promote immune and inflammatory responses and contribute to allograft rejection.^5,9^ In the context of experimental lung transplant, we previously demonstrated HA fragments act as innate immune agonists to stimulate AR and promote alloimmunity.^10^ We also translated these findings to human disease and demonstrated HA accumulates in the lung fluid in recipients with CLAD. Others have shown fragmented HA accumulates in the lungs of rejecting mice due to decreased clearance by local lymphatics and found tissue HA may be elevated in the setting of AR.^11^

Despite the potential importance of HA in posttransplant AR and CLAD, very few studies have directly examined HA expression in the bronchoalveolar lavage fluid (BALF) in the setting of clinical AR; previous studies were from a much earlier era or included very small sample cohorts with inconsistent results.^12-14^ We analyzed a multicenter cohort of well-characterized lung recipients with >300 lung-fluid samples collected at the time of an allograft biopsy within the first posttransplant year to test the hypothesis that HA increases in the lung allograft fluid in association with AR. These analyses examining the relationship between lung-fluid HA and biopsy-proven AR also adjusted for factors that could potentially confound HA measures. Because lymphocyte accumulation has been observed in the BALF in association with AR and is an indicator of adaptive immune activation, we also examine the association between BALF HA level and the BALF lymphocyte count. Finally, given our prior work demonstrating a role for HA in the pathobiology of CLAD, we also explored the association between early posttransplant BALF HA levels and the development of CLAD.

## MATERIALS AND METHODS

### Cohort

The cohort consisted of 126 first adult lung recipients with 373 BALF samples. Patients were transplanted between December 2015 and October 2016. The cohort was drawn from the Clinical Trials in Organ Transplantation-20 (CTOT-20) study, a prospective observational study collecting serial clinical data and biologic samples on newly transplanted patients at 5 North American centers. The CTOT-20 BALF collection and processing protocols are detailed in the supplement, addressing the elements of minimum required information for BAL-focused papers according to a recent International Society for Heart and Lung Transplant consensus statement.^15^ Samples included in the current analysis were collected within the first-year posttransplant and paired with a lung-tissue biopsy from the same bronchoscopy. Samples were excluded if collected on the same date as a biopsy ungradable for AR (Ax), or if collected on or after the onset of CLAD. The study was approved by the institutional review board (Pro00079551).

### Assays

BALF HA was quantified by commercial ELISA (R&D Systems; Minneapolis, MN). Samples falling below the lower limit of detection (n = 8) were assigned an HA concentration of the lowest detectable value on the plate.

### Clinical Assessments

Patients were managed according to center-specific clinical practice protocols as detailed in the supplement. Serial clinical data were prospectively collected in the CTOT-20 database. Data of interest included recipient age at transplant, body mass index, sex, race, native lung disease, transplant type, maintenance immunosuppresion at the time of posttransplant hospital discharge, and the occurrence of primary graft dysfunction grade 3 at 48 or 72 hours postreperfusion. Primary graft dysfunction grade was evaluated at the enrolling site according to International Society for Heart and Lung Transplantation guidelines.^16^ All BALF specimens were assessed as part of clinical care for culture growth of any bacterial, fungal, or mycobacterial organisms and for the presence of community acquired respiratory viral pathogens by polymerase chain reaction or other center-specific methodologies. For the majority of the BALF samples (328 of 373, 87.9%), the cellular composition of the sample was also assessed as part of clinical care and the percentage of each major cell type recorded in the database. The presence and severity of AR were determined on each biopsy according to the center pathologist using International Society for Heart and Lung Transplantation consensus guidelines.^17^ The presence of non-AR histologies on the lung biopsy specimen was also noted. CLAD was defined as a sustained ≥20% decline in the forced expiratory volume in 1 second (FEV1) as compared to the average of the 2 best posttransplant FEV1s measured at least 3 weeks apart in the absence of other clinical confounders.^18^

### Statistical Analyses

Descripive statistics were used to summarize cohort demographics and sample characteristics. Continuous variables were described with SD or range of the first and third quartiles (Q1, Q3) given to indicate the variability around the mean or median, respectively. Categorical variables were described with counts (proportions). For the descriptive analyses, biopsies were binned into the following groups based on timing of measurement posttransplant: 20–60 days/61–140 days/141–200 days/>200 days (roughly windowing 1, 3, 6, and 9 mo posttransplant). The distribution of BALF HA levels were reported across all samples and also stratified by AR status and by transplant type.

To test whether BALF HA is increased in lung recipients at the time of an AR event, BALF HA levels were modeled as a function of AR status and time from transplant using a linear-mixed effects model (LMM) with a random intercept and a random slope on time. Time was centered and scaled to ensure that large values do not overly influence the regression results. A LMM was used to account for dependencies in the observations. The model was adjusted for factors that could confound the association between BALF HA levels and AR including concurrent non-AR histological findings, concurrent infection, transplant type, and BAL instillation volume. Concurrent other histology was considered as the presence of any non-AR histology noted on the biopsy in addition to AR. Concurrent infection was considered as a positive BALF culture or polymerase chain reaction for any bacteria, fungus, mycobacteria, or virus at the time of the AR event. A sensitivity analysis was performed to understand if enrolling center influenced the association between AR and BALF HA levels (Supplemental Methods, SDC, http://links.lww.com/TP/C161). The BALF HA data were transformed using the natural-log to better meet normality assumptions for the inferential analyses.

To examine the association between BALF HA level and the BALF lymphocyte count, we included %-BALF lymphocyte count as a covariate in the LMM model. Because of the strong right skew in the observed distribution of the %-BALF lymphocyte count, the %-lymphocyte count was log-transformed before being entered into the model. An offset of 0.5 was also added to avoid log-transforming 0 values. Both an unadjusted model, which included log-transformed %-BALF lymphocyte count and time of sample collection posttransplant, and an adjusted model, which included all of the above-listed covariates were fit.

We next performed analyses to understand whether BALF HA levels correlate with resolution or persistence of AR in a subset of patients who experienced AR and for whom BALF HA was also determined on a subsequent biopsy (N = 56 patients). An ANCOVA model was fit to evaluate the change in BALF HA levels among the index and follow-up biopsy. Specifically, the ANCOVA modeled the BALF HA concentration at the time of the follow-up biopsy as a function of a binary indicator of AR resolution (yes versus no) and the BALF HA concentration at the time of the index biopsy demonstrating AR. The model was adjusted for the time between the index and follow-up biopsies and instillation volume of the follow-up BAL.

Last, we explored the association between posttransplant BALF HA levels measured within the first 6 months posttransplant and the development of CLAD after that time. We first identified the maximum BALF HA value (raw value in ng/mL) observed within the first 6 months of transplant for each subject. Subjects were then divided into tertiles based on this maximum observed HA value, with the first tertile representing those patients with the lowest maximum BALF HA levels and the third tertile representing those with the highest BALF HA levels. The cumulative incidence of CLAD, occurring from 6 months posttransplant to last follow-up before database lock in March 2020, was compared across the tertiles of maximum BALF HA level using the log-rank test.

## RESULTS

### Patient and Sample Characteristics

**FIGURE 1.**
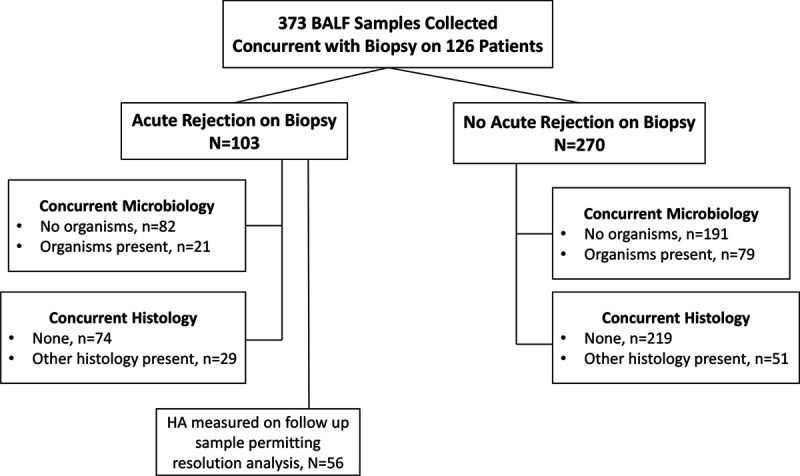
Description of included bronchoalveolar lavage fluid samples. BALF, bronchoalveolar lavage fluid; HA, hyaluronan.

The characteristics of the analysis cohort are detailed in Table 1 and a description of the included BALF samples is illustrated in Figure 1. The cohort was predominantly white with the majority (86 of 126, 68.3%) being male. Over half (69 of 126, 54.8%) had native restrictive lung disease and most (94 of 126, 74.6%) underwent bilateral lung transplantation. At the time of hospital discharge, all patients were on a calcineurin inhibitor for immunosuppression with the majority taking tacrolimus and nearly all (122 of 126, 96.8%) were also on a cell cycle inhibitor. The 126 patient study cohort contributed 373 BALF samples with the majority being collected in either the 1- or 3-month posttransplant window (Table S1, SDC, http://links.lww.com/TP/C161). The distribution of patients and samples among the 5 enrolling centers is detailed in Table S2 (SDC, http://links.lww.com/TP/C161). Of the 373 BAL analyzed samples, 332 (89%) were collected during routine surveillance bronchoscopy, whereas 41 (11%) were collected during a clinically indicated bronchoscopy. Examining the biopsies that paired with the 373 BALF samples, there were a total of 103 biopsy-proven AR events with 71 minimal (A1), 31 mild (A2), and 1 moderate (A3) in severity. The remaining 270 biopsies did not reveal histological evidence of AR (A0). The majority of BALF samples did not have concurrent infection and most had no concurrent non-AR histology (73.2% and 78.6%, respectively) (Table S3, SDC, http://links.lww.com/TP/C161).

**TABLE 1.** Patient characteristics overall and stratified by whether they contributed at least 1 acute rejection event to the analysis

	All patients with samples(N = 126)	Had at least 1 acute rejection event(N = 72)	Did not have an acute rejection event(N = 54)	*P* ^* a*^
Collected at time of transplant				
Age at transplant (y)	60.0 (51.0, 67.0)	60.0 (54.0, 67.2)	60.0 (48.8, 67.0)	0.60
BMI	25.2 (22.1, 27.6)	25.1 (22.2, 27.6)	25.3 (21.8, 27.7)	0.99
Female	40 (31.7%)	24 (33.3%)	16 (29.6%)	0.70
Race				0.37
White	110 (87.3%)	65 (90.3%)	45 (83.3%)	
Black	9 (7.1%)	3 (4.2%)	6 (11.1%)	
Other	7 (5.6%)	4 (5.6%)	3 (5.6%)	
Primary disease				0.19
Restrictive	69 (54.8%)	45 (62.5%)	24 (44.4%)	
Obstructive	38 (30.2%)	16 (22.2%)	22 (40.7%)	
Cystic fibrosis	13 (10.3%)	7 (9.7%)	6 (11.1%)	
Vascular	3 (2.4%)	2 (2.8%)	1 (1.9%)	
Other	3 (2.4%)	2 (2.8%)	1 (1.9%)	
Transplant type				0.31
Single	32 (25.4%)	21 (29.2%)	11 (20.4%)	
Bilateral	94 (74.6%)	51 (70.8%)	43 (79.6%)	
Collected posttransplant				
PGD grade 3 at 48 h or 72 h	18 (14.3%)	8 (11.1%)	10 (18.5%)	0.31
Primary maintenance immunosuppression				0.99
Cyclosporine	9 (7.1%)	5 (6.9%)	4 (7.4%)	
Tacrolimus	118 (93.7%)	67 (93.1%)	51 (94.4%)	
Cell cycle inhibitor, yes	122 (96.8%)	69 (95.8%)	53 (98.1%)	0.64
Induction immunosuppression, yes	72 (57.1%)	36 (50.0%)	36 (66.7%)	0.07

Summary measures are median (25th percentile, 75th percentile) for continuous variables and are count (proportion) for categorical variables.

^*a*^Continuous variables compared between patients that had at least 1 acute rejection vs those that did not have acute rejection using Wilcoxon rank sum test and Fisher’s exact test for categorical variables.

BMI, body mass index; PGD, primary graft dysfunction.

### Distribution of HA Concentration in the BALF

**FIGURE 2.**
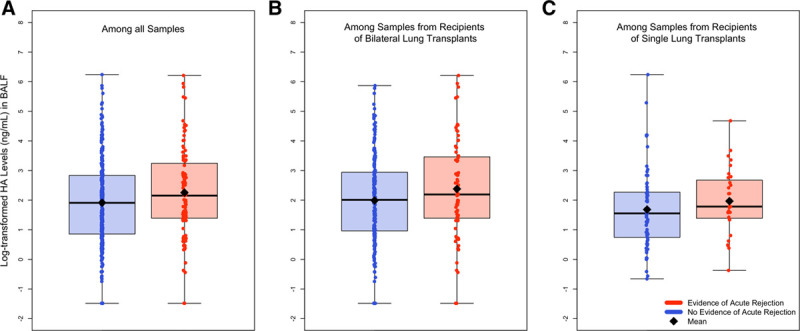
Distribution of log-transformed BALF HA concentrations by acute rejection status among all available samples (A) and as stratified by bilateral (B) or single (C) lung transplant recipient. BALF, bronchoalveolar lavage fluid; HA, hyaluronan.

In descriptive analyses considering all samples, we observed a nonnormal distribution of BALF HA concentrations with a median (Q1, Q3) of 7.26 (2.58, 17.71) ng/mL and a mean (SD) of 24.89 (59.85) ng/mL (Table 2). Bilateral lung recipients had numerically higher median BALF HA values than single lung recipients (7.83 versus 5.16 ng/mL). We also observed higher median HA values in BALF samples with larger BAL instillation volumes (Table S3, SDC, http://links.lww.com/TP/C161), although the median instillation volume was identical among samples with versus those without AR (80.0 versus 80.0 mL). The distribution of BALF HA concentration was similar among samples obtained during surveillance as compared with clinically indicated bronchoscopies (Figure S1, SDC, http://links.lww.com/TP/C161). Notably, BALF samples that paired with a biopsy demonstrating histological evidence of AR had higher median HA concentrations than those pairing with biopsies not demonstrating AR histology (8.61 versus 6.75 ng/mL) (Table 2; Figure S2, SDC, http://links.lww.com/TP/C161). This trend was similar in both bilateral and single lung recipients (Table S4, SDC, http://links.lww.com/TP/C161). Overall similar patterns in the distribution of the BALF HA levels were observed on the log-scale. The distribution of the log-transformed BALF HA concentrations stratified by AR status on the paired biopsy in all samples and as subset by transplant type are represented in Figure 2.

**TABLE 2.** Distribution of HA concentrations by acute rejection status in the BALF among all available samples

	By AR status		
	Among all samples(N = 373)	Samples with AR on biopsy(N = 103)	Samples without AR on biopsy(N = 270)
BALF HA concentration (ng/mL)			
Range (minimum, maximum)	0.23, 512.13	0.23, 497.18	0.23, 512.13
Median (Q1, Q3)	7.26 (2.58, 17.71)	8.61 (4.02, 25.69)	6.75 (2.37, 16.88)
Mean (SD)	24.89 (59.85)	33.16 (76.09)	21.73 (52.18)

AR, acute rejection; BALF, bronchoalveolar lavage fluid; HA, hyaluronan.

### Association of BALF HA With Biopsy-proven AR or BALF Lymphocytes

As patients in this cohort contributed multiple samples to the analysis, we next fit an LMM to test whether BALF HA is increased in lung transplant recipients at the time of biopsy-proven AR. In unadjusted analyses, we observed a significant association between BALF HA and biopsy AR status with a mean increase in BALF HA of 0.37 units on the log-scale at the time of an AR event (change in means on log-scale 0.37; 95% CI, 0.09-0.66; *P* = 0.011) (Table 3). The results were similar when adjusting for potential confounders including timing of sample collection posttransplant, concomitant infection, non-AR histology, type of transplant, and BAL instillation volume (change in means on log-scale 0.31; 95% CI, 0.01-0.60; *P* = 0.044). When considered on the original scale (ng/mL), these results suggest BALF HA concentrations are 1.36 times (or 36%) higher, on average, among samples with, versus without, AR. These observations were similar in a sensitivity analysis adjusting for enrolling center (Table S5, SDC, http://links.lww.com/TP/C161).

Interestingly, there was no significant unadjusted or adjusted association between BALF HA levels and concomitant infection or non-AR histology suggesting HA may be specific for AR. We did, however, observe a significant association between BALF HA concentration and BAL instillation volume in both unadjusted and adjusted analyses (Table 3), with larger BAL instillation volumes being associated with higher BALF HA levels (adjusted change in means on log-scale 0.87; 95% CI, 0.31-1.42; *P* = 0.002). Additionally, there was a significant association between BALF HA concentration and time posttransplant in both unadjusted and adjusted analyses with a mean decrease of −0.23 units on the log-scale per 1 SD of time (adjusted difference in means on log-scale −0.23 for every 69 d; 95% CI, −0.39 to −0.08; *P* = 0.003). These results indicate BALF HA decreases with time elapsed after transplant, a finding further illustrated in Figure S3 (SDC, http://links.lww.com/TP/C161). To understand whether the relationship between AR and HA levels varied by time between transplant and sample collection and or by type of transplant (single versus bilateral), we individually tested for an interaction between time and AR and between transplant type and AR. We did not observe a significant interaction (*P* = 0.348 and *P* = 0.851, respectively), suggesting the relationship between AR and HA remains the same over time and regardless of transplant type (Table 3).

**TABLE 3.** Association between BALF HA concentration and AR status on biopsy among all available samples

	Unadjusted^*a*^		Adjusted^*a*^	
	Effect estimate (95% CI)	*P*	Effect estimate (95% CI)	*P*
Main effects				
AR		0.011		0.044
Additive change in means on the log-scale^*b*^	0.37 (0.09, 0.66)		0.31 (0.01, 0.60)	
Multiplicative change in means on the original-scale^*b*^	1.45 (1.09, 1.93)		1.36 (1.01, 1.83)	
Concomitant infection		0.809		0.761
Additive change in means on the log-scale	0.04 (−0.26, 0.33)		0.05 (−0.25, 0.34)	
Multiplicative change in means on the original scale	1.04 (0.77, 1.39)		1.05 (0.78, 1.41)	
Other concomitant histology		0.167		0.191
Additive change in means on the log-scale	0.21 (−0.09, 0.52)		0.21 (−0.10, 0.52)	
Multiplicative change in means on the original scale	1.24 (0.91, 1.68)		1.23 (0.90, 1.68)	
Bilateral transplant		0.292		0.145
Additive change in means on the log-scale	0.26 (−0.22, 0.74)		0.35 (−0.12, 0.83)	
Multiplicative change in means on the original scale	1.29 (0.80, 2.09)		1.42 (0.88, 2.29)	
BAL instillation volume (log-mL)		0.002		0.002
Additive change in means on the log-scale	0.86 (0.32, 1.40)		0.87 (0.31, 1.42)	
Multiplicative change in means on the original scale	2.37 (1.38, 4.07)		2.38 (1.37, 4.13)	
Time of sample collection (per 1 SD = 69 d)		0.001		0.003
Additive change in means on the log-scale	−0.27 (−0.42, −0.11)		−0.23 (−0.39, −0.08)	
Multiplicative change in means on the original scale	0.77 (0.66, 0.89)		0.79 (0.68, 0.92)	
AR effect stratified by time of sample collection^*c*^				
Collected 1 mo posttransplant		0.019		0.035
Additive change in means on the log-scale	0.45 (0.08, 0.83)		0.42 (0.03, 0.81)	
Multiplicative change in means on the original scale	1.58 (1.08, 2.30)		1.52 (1.03, 2.26)	
Collected 3 mo posttransplant		0.012		0.045
Additive change in means on the log-scale	0.37 (0.08, 0.66)		0.30 (0.01, 0.60)	
Multiplicative change in means on the original scale	1.45 (1.09, 1.93)		1.36 (1.01, 1.83)	
AR effect stratified by transplant type^*d*^				
Among single lung transplants		0.194		0.389
Additive change in means on the log-scale	0.34 (−0.18, 0.86)		0.24 (−0.31, 0.79)	
Multiplicative change in means on the original scale	1.41 (0.84, 2.36)		1.27 (0.73, 2.20)	
Among bilateral lung transplants		0.026		0.062
Additive change in means on the log-scale	0.39 (0.05, 0.73)		0.33 (−0.02, 0.68)	
Multiplicative change in means on the original scale	1.47 (1.05, 2.07)		1.39 (0.98, 1.97)	

^*a*^Unadjusted models include each covariate individually and time of sample collection; adjusted models include all covariates simultaneously.

^*b*^The additive and multiplicative change estimates for each covariate are derived from the same statistical model and express the observed findings both on the log and on the original scale, respectively, for ease of interpretation.

^*c*^The *P* value of the interaction test for acute rejection status and time of sample collection is 0.348.

^*d*^The *P* value of the interaction test for acute rejection status and transplant type is 0.851.

AR, acute rejection; BALF, bronchoalveolar lavage fluid; CI, confidence interval; HA, hyaluronan.

We next examined the association between BALF HA level and the percentage of lymphocytes in the BALF by including the %-BALF lymphocyte count as a covariate in the LMM. Notably, both the unadjusted and adjusted models indicated a significant association between %-BALF lymphocytes and BALF HA levels, with higher HA concentrations observed among samples with higher %-lymphocyte counts (adjusted difference in means on log-scale 0.18; 95% CI, 0.06-0.30; *P* = 0.004) (Table S6, SDC, http://links.lww.com/TP/C161).

### Association of BALF HA With AR Resolution or Persistence

**FIGURE 3.**
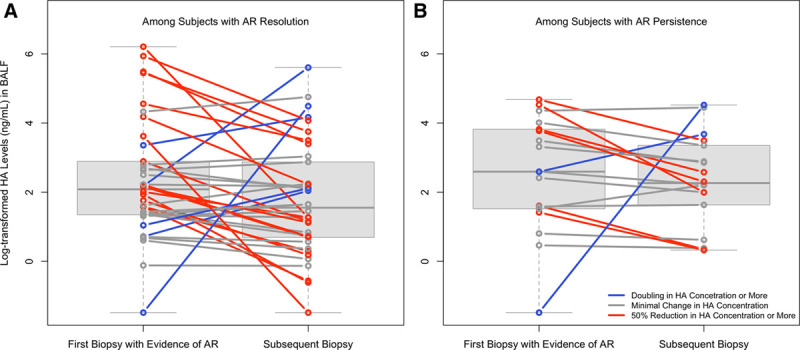
Distribution of log-transformed BALF HA concentration by acute rejection resolution (A) or persistence (B) between the index and follow-up biopsy. AR, acute perivascular rejection; BALF, bronchoalveolar lavage fluid; HA, hyaluronan.

We examined whether BALF HA levels correlate with AR resolution or persistence. The median (Q1, Q3) time between the index and follow-up biopsy was 36.0 (27.0, 49.5) days. Table S5 (SDC, http://links.lww.com/TP/C161) and Figure 3 describe the distribution of index and follow-up BALF HA levels in these 56 patients stratified by whether AR was resolved or persistent on follow-up biopsy. In unadjusted analyses and in analyses adjusted for the time between biopsies, BALF HA concentration at the time of the index biopsy, and BAL instillation volume, patients with AR resolution had a greater numeric decline in BALF HA versus those with AR persistence; however, this result was not statistically significant (unadjusted change in mean log-HA −0.49; 95% CI, −1.33-0.34; *P* = 0.243, adjusted change in mean log-HA −0.70; 95% CI, −1.52-0.11, *P* = 0.089). When considered on the original scale (ng/mL), these results suggest BALF HA concentrations were 0.49 times (or 49%) lower, on average, among follow-up samples corresponding to AR resolution versus persistence.

### Relationship Between Early Posttransplant BALF HA and Development of CLAD

**FIGURE 4.**
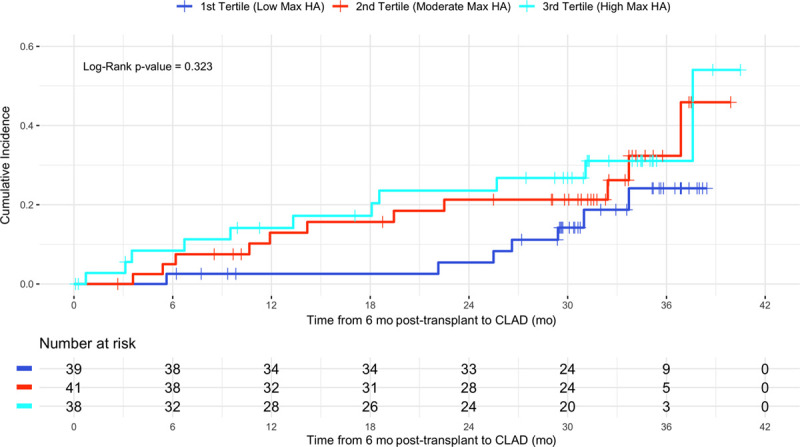
Cumulative incidence of CLAD by strata of maximum HA concentration observed in the bronchoalveolar lavage fluid within the first 6 mo of lung transplantation. Tertile cutpoints were as follows—tertile 1 HA levels 1.6750–7.9214 ng/mL, tertile 2 HA levels 7.9215–31.3980 ng/mL, tertile 3 HA levels >31.3980 ng/mL. Thus, tertile 1 represents thus subjects with the lowest maximum observed HA levels in the first 6 mo posttransplant, whereas tertile 3 represents those subjects with the highest maximum observed HA levels in the first 6 mo posttransplant. CLAD, chronic lung allograft dysfunction; HA, hyaluronan.

Thirty-one patients developed CLAD over follow-up (31 of 126, 24.6%) with 29 of these CLAD events occurring after 6-month posttransplant. The cumulative incidence of CLAD was numerically higher in patients in the highest tertiles of maximum BALF HA level within the first 6 months after transplant, as compared with those in the lowest tertile of maximum HA level (Figure 4). Although a numerical trend was observed, this difference was not statistically significant (*P* = 0.32).

## DISCUSSION

We determined the relationship between BALF HA concentration and AR in the early posttransplant period in a multicenter cohort of lung transplant recipients, including >300 BALF samples and >100 biopsy-proven AR events. Our results demonstrate that AR is associated with an increase in BALF HA levels. Specifically, BALF HA concentrations were 36% higher on average, among samples with, versus without, AR. Moreover, increased BALF HA may be specific for AR, as HA concentrations did not appear to be significantly influenced by concurrent infection or other non-AR histologies on biopsy. Consistent with this idea, in a subset of patients who experienced AR, there was a trend toward reduction in BALF HA concentration with AR resolution.

Very few studies have examined HA in AR.^12-14^ First, Riise et al investigated BALF HA concentrations in 19 patients with 54 AR episodes.^14^ Although the authors determined mean BALF HA level was higher during AR, this result did not remain significant when accounting for repeat measures in the same individual, timing of sample collection posttransplant, and concomitant infection.^14^ In a more recent single-center study, Courtright et al examined HA concentrations in the blood and BALF in 115 samples from 78 patients with 27 AR events to report no association between either serum or BALF HA and AR.^13^ Although many factors could have contributed to divergence in the results of our analysis with those from this prior work, it is likely that the much larger number of AR events under consideration in our analysis supported improved statistical power. Additionally, the latter study did not appear to apply a mixed modeling strategy, which permits the use of data from serially sampled individuals while accounting for the lack of independence.

The association between HA accumulation in the lung allograft and AR could result from excess production, decreased clearance, or both. Although little is known regarding HA synthesis in the context of AR, prior work suggests HA clearance alterations in experimental lung transplantation. Specifically, in a preclinical lung transplant model, HA accumulates in murine lung AR,^11^ results entirely consistent with our clinical study. Moreover, this prior preclinical study showed HA accumulation is due to impaired HA clearance through pulmonary lymphatics via the lymphatic vessel endothelial receptor-1.^11^ Importantly, murine AR was diminished after therapeutic lymphangiogenesis.^11^ In addition to functions in interstitial fluid drainage and immune cell transport, the lymphatics account for about 85% of HA turnover in the lung through lymphatic vessel endothelial receptor-1 expression on lymphatic endothelial cells.^19^ As the transplant operation is known to disrupt pulmonary lymphatics, this preclinical work suggests a plausible mechanism by which HA could accumulate in AR.

The implications of accumulation of HA during AR on pulmonary inflammation are suggested in work by Noble et al.^20^ In this prior study, the authors used a model of bleomycin-induced lung injury to demonstrate HA clearance is required to successfully resolve pulmonary inflammation. In particular, they showed mice genetically deficient in the HA clearance receptor CD44 developed far more pulmonary inflammation and prolonged proinflammatory cytokine induction when compared with wild-type mice. This unremitting inflammation was accompanied by a continued rise in lung HA content with marked intrapulmonary HA accumulation and respiratory failure in CD44 deficient mice.^20^ The results of Noble et al are of particular interest given our prior observations of HA accumulation in the BALF, blood, and tissue in lung recipients with CLAD.^10^ In this prior work, we found recipients with CLAD have a far greater mean BALF HA content (107.91 ng/mL) than that observed with AR (24.89 ng/mL) in the current study. Together, these findings support the idea that HA plays a critical role in the spectrum from acute inflammation, such as occurs in AR, and persistently altered immune responses and extracellular matrix deposition that contribute to CLAD.

The observation of increased HA in AR and CLAD in human-lung recipients is also consistent with our previous work in which we demonstrated HA regulates intragraft immune responses in murine lung transplantation.^10^ Using preclinical models, we demonstrated HA, specifically low molecular–weight HA fragments, stimulated acute allograft rejection in murine lung recipients with established tolerance, a response dependent on innate immune activation via the toll-like receptor 4. Notably, the rejection response invoked by HA was accompanied not only by an increase in IFN-y^+^CD4^+^ and IFN-y^+^CD8^+^ T-cells but also by intragraft IL-17^+^CD4^+^ T-cell accumulation,^10^ suggesting HA is able to disrupt intragraft regulatory mechanisms and promote differentiation of effector T-cell populations.^10^ The data we report herein, indicating a relationship between BALF HA levels and BALF lymphocyte count in human-lung recipients, lend new perspective to these experimental findings and suggest HA may facilitate innate-adaptive crosstalk in human-lung recipients.

Taken together this body of prior work suggests AR leads to increased production of HA that is further exacerbated by impaired HA clearance through graft lymphatics. Failure to clear HA matrix then further potentiates the alloimmune response. Moreover, persistence of alloimmune injury may then also contribute to further HA accumulation, thus inciting a cycle of sustained inflammation and extracellular matrix deposition. This idea is supported by our findings of a trend toward persistently increased BALF HA levels in nonresolving AR, which may reflect an intermediate clinical state in the progression from AR to CLAD, and further bolstered by our finding that early posttransplant elevations in BALF HA correspond to a numerical increase in the cumulative incidence of CLAD. The observations herein, in addition to our prior observations demonstrating increased HA expression in CLAD, provide a rationale for future clinical studies to understand how early posttransplant elevations in BALF HA influence longer-term graft outcomes. Moreover, these observations compel further mechanistic investigations to define the effects of HA on immune cell function within the human-lung allograft and develop approaches to reduce HA accumulation as a novel strategy to attenuate development of CLAD.

 the strengths of our study include its multicenter and translational design, a substantive number of biopsy-proven AR events under consideration, and statistical modeling that took into account the repeated sampling in the cohort in addition to potential confounding variables, there are limitations. First, our study included only BALF samples obtained in the early posttransplant period with most samples taken within the first ~3 months posttransplant. Therefore, further validation of our findings with longitudinal characterization of BALF HA is necessary to permit a comprehensive understanding of HA dynamics posttransplant. Additionally, our analyses focused only on perivascular acute cellular rejection given the infrequency of lymphocytic bronchiolitis events (n = 18) in this cohort. Second, we may have been underpowered to detect an association between BALF HA levels and concurrent infection, given less than one-quarter of our included samples corresponded to an infectious event. Similarly, our analyses focused on BALF HA and AR resolution versus persistence or CLAD risk were likely underpowered given the smaller number of events under consideration. Finally, HA molecular size has proven difficult to examine in previous experimental and human studies and despite attempts with agarose gel electrophoresis, we were unable to directly assess BALF HA molecular size.

In conclusion, we found increases in BALF HA in human-lung recipients with AR. This novel observation lends clinical relevance to a foundation of mechanistic insights regarding the influence of HA on the pulmonary alloimmune response. Detailed translational studies to clarify the cell types contributing to HA synthesis or the abundance and distribution of HA clearance receptors in the lung allograft will refine our understanding of the pathways promoting HA accumulation in clinical AR. Additionally, ongoing serial data collection on this cohort will permit future, well-powered analyses examining how early posttransplant elevations in BALF HA influence the risk for CLAD development. Such translational and clinical studies are critical to fully inform the biologic spectrum from AR to CLAD in lung transplant recipients and develop new approaches to improve long-term patient outcomes.

## Supplementary Material


