# Supplementary material for: Intragraft Hyaluronan Increases in Association With Acute Lung Transplant Rejection
Source: Transplant Direct. 2021 Mar 22;7(4):e685. doi: 10.1097/TXD.0000000000001138 (PMC8440013; doi:10.1097/TXD.0000000000001138)
Supplement: Supplementary file 1 [file txd-7-e685-s001.pdf]

Supplemental Material

Table of Contents:

Supplemental Methods: Pages 2-9

Supplemental Figures: Page 10-13

Supplemental Tables: Pages 14-19

SUPPLEMENTAL MATERIAL

Methods

Collection and processing protocols for lung fluid samples:

In order to retrieve bronchoalveolar lavage fluid (BALF) for this study the recommended protocol was to instill and withdraw two 50-60mL aliquots of sterile normal saline into the right middle lobe or lingula. Further aliquots could be performed at the discretion of the bronchoscopist until the total volume returned was adequate to perform the necessary testing. In the event the patient was tolerating the procedure poorly or the return volume from the first aliquot was sufficient for clinical and research purposes, the second aliquot could be deferred at the bronchoscopist's discretion. Samples from other lobar locations were acceptable if that was the only lobe targeted for clinical collection. Return samples were pooled and labeled with the subsegmental location. A quantity sufficient for standard clinical testing (generally ~15 mL) was sent to the clinical laboratory and the remainder, 5 mL (minimum) to 25 mL (maximum), was sent for the research study. For each bronchoscopy, both the BAL instillation and return volumes were recorded in the central database. BALF samples were stored on ice or at 4°C while awaiting the start of processing, and between all processing steps. BALF specimens were processed within 4 hours of collection. To separate the BALF supernatant (the matrix of interest for this study) from the cellular component, the BALF was put through a 100μm cell strainer into a 50mL sterile tube then the filtered sample centrifuged at 500g for 10 minutes at 4-10°C using a swinging bucket rotor. The supernatant was then aliquoted into cryovials and stored at -70°C to -80°C until analysis.

Sensitivity analysis to account for enrolling center effects

To determine if enrolling center influenced the observed association between AR and BALF HA levels, we performed a sensitivity analysis adjusting the linear mixed effects model for enrolling center. Because center and BAL volume instilled were observed to be strongly correlated (*p* value < 0.001), for the sensitivity analysis volume instilled was removed from the list of covariates and replaced with center. In this way, the sensitivity analysis controls for all potential heterogeneity due to enrolling center, including differences in BAL volume instilled.

General Clinical Management Practices at the 5 Enrolling Centers

Duke University: Immunosuppression is induced with methylprednisolone and 2 doses of basiliximab. Thereafter, maintenance immunosuppression includes prednisone, mycophenolate mofetil and tacrolimus. For patients intolerant of mycophenolate mofetil, azathioprine is substituted. For patients intolerant of tacrolimus, cyclosporine is substituted. Within the first posttransplant year, surveillance bronchoscopies are performed routinely at 1, 3, 6, 9, and 12 months with additional bronchoscopies as clinically indicated. Routine microbiological studies sent from each bronchoscopy include bacterial, fungal, and AFB cultures in addition to respiratory viral PCR multiplex. Acute rejection or lymphocytic bronchiolitis is treated with methylprednisolone for initial episode and minimal or mild acute rejection. Recurrent or refractory acute rejection or lymphocytic bronchiolitis is treated with antithymocyte globulin or alemtuzumab. Patients treated for acute rejection or lymphocytic bronchiolitis typically are rebiopsied at 4-6 week intervals until 2 consecutive biopsies demonstrate histological resolution. All patients are

monitored serially for the development of anti-HLAs including any donor specific antibodies (DSAs) at minimum at the time of each posttransplant bronchoscopy. Those patients with a history of DSA have additional screening at each posttranplant clinic visit until antibody resolution. Patients with CMV mismatch (D+/R-) are managed with valganciclovir for the lifetime of the allograft, intermediate risk patients (R+) are managed with valganciclovir for the first posttransplant year, low risk patients (D-/R-) receive 3 months of acyclovir. All lung recipients receive fungal prophylaxis with fluconazole for the first 3 months after transplantation. All lung recipients receive pneumocystis prophylaxis with trimethoprim/sulfamethoxazole or other targeted agent.

University of California Los Angeles: Induction immunosuppression includes methylprednisolone in addition to either antithymocyte globulin 1.5mg/kg given on post operative days 0, 1 and 2 for those under the age of 60 years or 2 doses of basiliximab given on days 0 and 4 for those aged 60 or greater. Maintenance immunosuppression is with tacrolimus, steroids, and mycophenolate mofetil. For patients intolerant of mycophenolate mofetil, mycophenolic acid or azathioprine is substituted. For patients intolerant of tacrolimus, cyclosporine is substituted. Within the first posttransplant year, surveillance bronchoscopies are performed routinely at 1 week (BAL only), and for BAL and biopsy at 1, 3, 6, and 12 months with additional bronchoscopies as clinically indicated. Routine microbiological studies sent from each bronchoscopy include bacterial, fungal, and AFB cultures in addition to respiratory viral PCR multiplex in addition to pneumocystis direct detection, CMV PCR, legionella culture, and mycoplasma PCR. Acute rejection grade A1, which is not accompanied by clinical signs or symptoms is treated with an oral

steroid taper. Symptomatic A1 rejection or rejection of grade A2 or higher is treated with IV methylprednisolone. Recurrent or refractory acute rejection is treated with a second pulse of IV methyprednisolone or antithymocyte globulin. Patients treated for acute rejection are typically not rebiopsied unless there are persistent or recurrent signs or symptoms of graft dysfunction. LB that is not accompanied by clinical signs or symptoms is not typically treated with augmented immunosuppression and is not rebiopsied. All patients are monitored serially for the development of anti-HLAs including any donor specific antibodies (DSAs) at 1, 3, 6, 9 and 12 months posttransplant and annually thereafter. Those patients with a history of DSA have additional screening at each posttranplant clinic visit until antibody resolution. Patients are managed with valganciclovir for the lifetime of the allograft regardless or recipient or donor CMV status. All lung recipients receive fungal prophylaxis with either voriconazole or posaconazole for 6 months in addition to inhaled amphotericin twice weekly for the duration of the postoperative hospitalization. All lung recipients receive pneumocystis prophylaxis with sulfamethoxazole and trimethoprim or other targeted agent.

University of Toronto: Induction immunosuppression over and above methylprednisolone is not routinely used in the absence of a positive crossmatch. If a positive crossmatch is detected antithymocyte globulin for a total of 3-5mg/kg is administered usually beginning on postoperative day 7. Maintenance immunosuppression is with cyclosporine, prednisone and azathioprine or mycophenolate (if recipient has any panel reactive antibodies at transplant, or if donor-specific antibodies subsequently develop). Cyclosporine is switched to tacrolimus and/or azathioprine is switched to mycophenolate for intolerance, recurrent/refractory acute rejection, or chronic lung allograft dysfunction.

Standardized program protocols are used for CNI target levels based on time since transplant, HLA antibody history and recipient age. Within the first posttransplant year, surveillance bronchoscopies are performed routinely at 2 weeks, 6 weeks, and at months 3, 6, 9, and 12 with additional bronchoscopies as clinically indicated. Routine microbiological studies sent from each bronchoscopy during the first postoperative year include bacterial, fungal, and AFB cultures in addition to respiratory viral PCR for influenza and respiratory syncytial virus, and galactomannan assay. Acute rejection or lymphocytic bronchiolitis of grade A1 or B1, respectively, which is not accompanied by clinical signs or symptoms is not treated with augmentation of immunosuppression though maintenance immunosuppression will be optimized if possible. Symptomatic grade 1 rejection or rejection of grade 2 or higher is treated with IV methylprednisolone and prednisone augmentation with taper. Recurrent or refractory acute rejection is treated with antithymocyte globulin. Patients treated for acute rejection or lymphocytic bronchiolitis typically are rebiopsied after prednisone taper. All patients are monitored serially for the development of anti-HLAs including any donor specific antibodies (DSA) at minimum at the time of each posttransplant bronchoscopy. Patients with CMV mismatch (D+/R-) are managed with valganciclovir for 9 months posttransplant, intermediate risk patients (R+) are managed with valganciclovir for 6 months posttransplant, low risk patients (D-/R-) receive acyclovir for 3 months posttransplant. Patients with recent pretransplant or intraoperative respiratory cultures positive for *Aspergillus* or galactomannan receive 3 months of voriconazole. All lung recipients receive pneumocystis prophylaxis with sulfamethoxazole and trimethoprim or other targeted agent.

Johns Hopkins University: Immunosuppression is induced with methylprednisolone and 2 doses of basiliximab. Thereafter, maintenance immunosuppression includes prednisone, mycophenolate mofetil and tacrolimus. For patients intolerant of mycophenolate mofetil, azathioprine is substituted. For patients intolerant of tacrolimus, cyclosporine is substituted. Within the first posttransplant year, surveillance bronchoscopies are performed routinely at 1, 3, 6, 9, and 12 months with additional bronchoscopies as clinically indicated. Routine microbiological studies sent from each bronchoscopy include bacterial, fungal, and AFB cultures in addition to respiratory viral PCR multiplex, direct fluorescence for pneumocystis and CMV early antigen testing. Acute rejection or lymphocytic bronchiolitis of grade A1 or B1, respectively, is treated with an oral steroid taper. More severe grades of rejection or rejection events of any grade associated with a significant spirometric decline are treated with IV methylprednisolone. Recurrent or refractory acute rejection or lymphocytic bronchiolitis is treated with IV methylprednisolone and with further augmentation individualized per patient.Follow up biopsies are usually conducted 3-6 weeks following detected AR, if safe to do so. All patients are monitored serially for the development of anti-HLAs including any donor specific antibodies (DSAs) at minimum at of every 3 months during the first posttransplant year, usually concurrent to bronchoscopy. Patients with CMV mismatch (D+/R-) are managed with valganciclovir for 6 months posttransplant, intermediate risk patients (R+) are managed with valganciclovir for 3 months posttransplant, low risk patients (D-/R-) receive lifelong acyclovir for varicella prophylaxis. Early postoperative fungal prophylaxis in all patients include inhaled amphotericin (for upto 6 weeks or hospital discharge) and oral nystatin, with a third generation azole used

in patients with a history of pretransplant *Aspergillus*. All lung recipients receive pneumocystis prophylaxis with trimethoprim/sulfamethoxazole or other targeted agent.

Cleveland Clinic: Induction immunosuppression over and above methylprednisolone is not routinely used in the absence of a positive crossmatch. In the setting of a positive crossmatch recipients are treated with antithymocyte globulin daily for 3 to 5 days in addition to steroids. Maintenance immunosuppression for all recipients includes tacrolimus, steroids, and mycophenolate mofetil. For patients intolerant of mycophenolate mofetil, azathioprine is substituted. For patients intolerant of tacrolimus, cyclosporine is substituted. Within the first posttransplant year, surveillance bronchoscopies are performed routinely at 3 weeks, 6 weeks, 3 months, 6 months, 9 months and 12 months with additional bronchoscopies as clinically indicated. Routine microbiological studies sent from each bronchoscopy include bacterial, fungal, and AFB cultures in addition to respiratory viral PCR multiplex, legionella PCR, *Pneumocystis Jiroveci* PCR, nocardia culture, aspergillus galactomannan and herpes simplex virus PCR. Acute rejection or lymphocytic bronchiolitis is treated with oral steroid taper for the initial episode of minimal or mild acute rejection. Recurrent or refractory acute rejection or lymphocytic bronchiolitis is treated with escalating doses of intravenous steroids or antithymocyte globulin. Patients treated for acute rejection or lymphocytic bronchiolitis are rebiopsied after 3 weeks to demonstrate histological resolution. All patients are monitored serially for the development of anti-HLAs including any donor specific antibodies (DSAs) starting on postoperative day 10 and subsequently at the time of each bronchoscopy during the first posttransplant year. Patients with CMV mismatch (D+/R-) and CMV intermediate risk patients (R+) are managed with valganciclovir for at least 1

year and longer if clinically well tolerated. CMV low risk patients (D-/R-) receive acyclovir for 1 year. All lung recipients receive fungal prophylaxis with oral itraconazole for 18 months posttransplant in addition to inhaled amphotericin twice weekly until itraconazole levels are therapeutic (serum level >0.4 uq/mL). All lung recipients receive pneumocystis prophylaxis with trimethoprim/sulfamethoxazole or other targeted agent.

SUPPLEMENTAL FIGURES

Figure S1: Distribution of raw (A) and log-transformed (B) BAL HA levels among for-cause clinically indicated bronchoscopies vs. surveillance bronchoscopies.

A

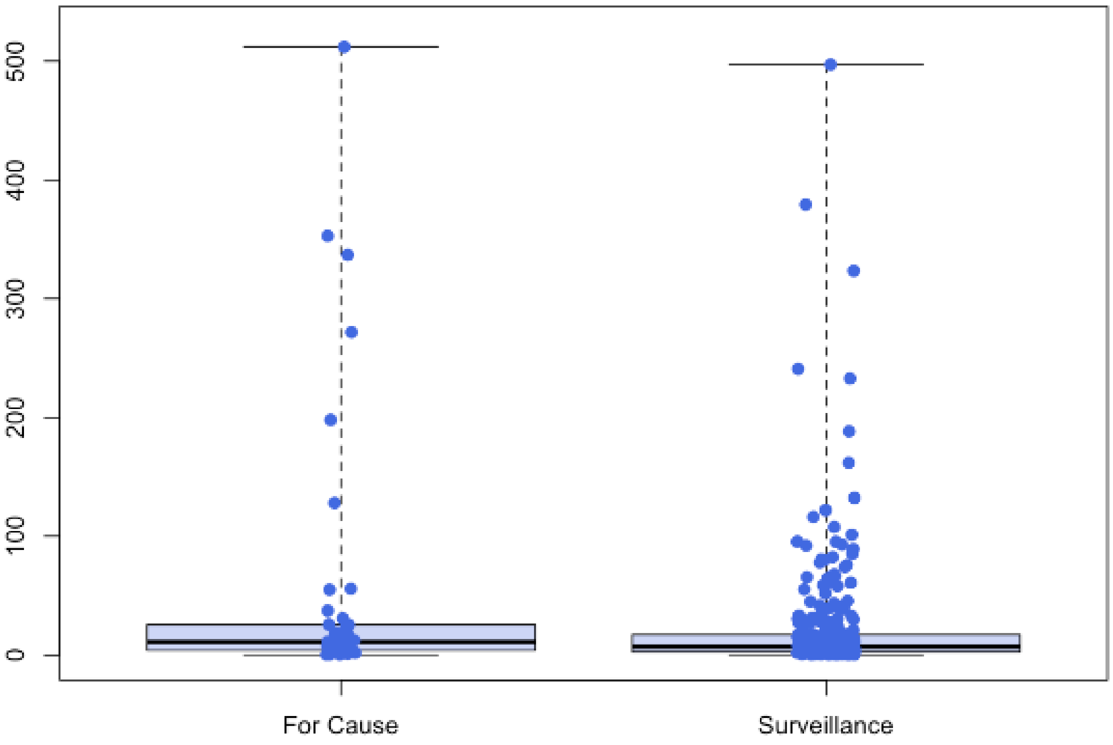

500
400
300
200
100
0
For Cause
Surveillance

B

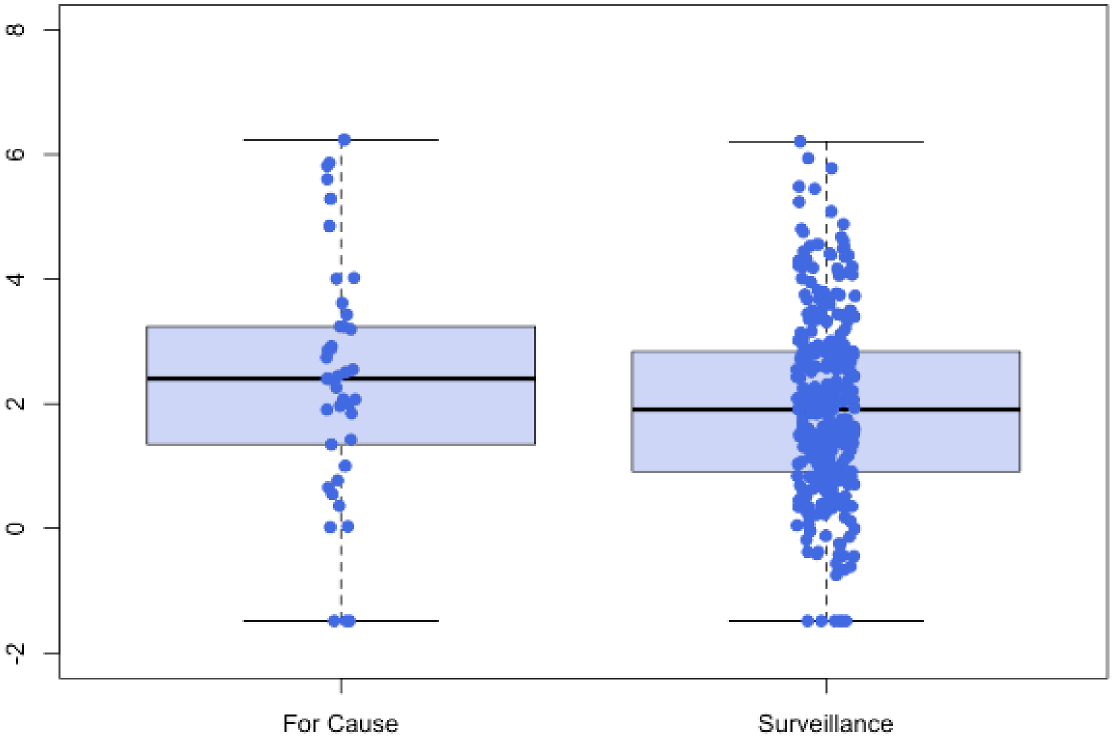

8
6
4
2
0
-2
For Cause
Surveillance

Figure S2. Distribution of HA concentrations (ng/mL) in the bronchoalveolar lavage fluid by acute rejection status among all available samples (A) and as stratified by bilateral (B) or single (C) lung transplant recipient.

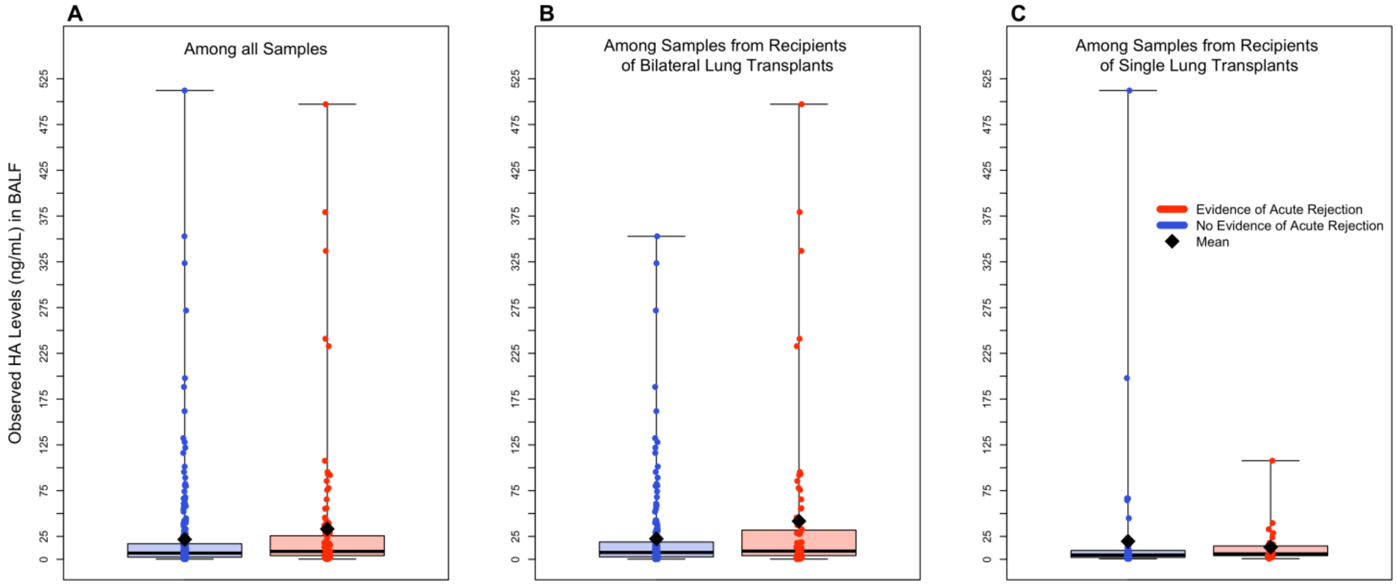

Figure S3: Log-transformed HA levels (ng/mL) over time in those with and without evidence of acute rejection.

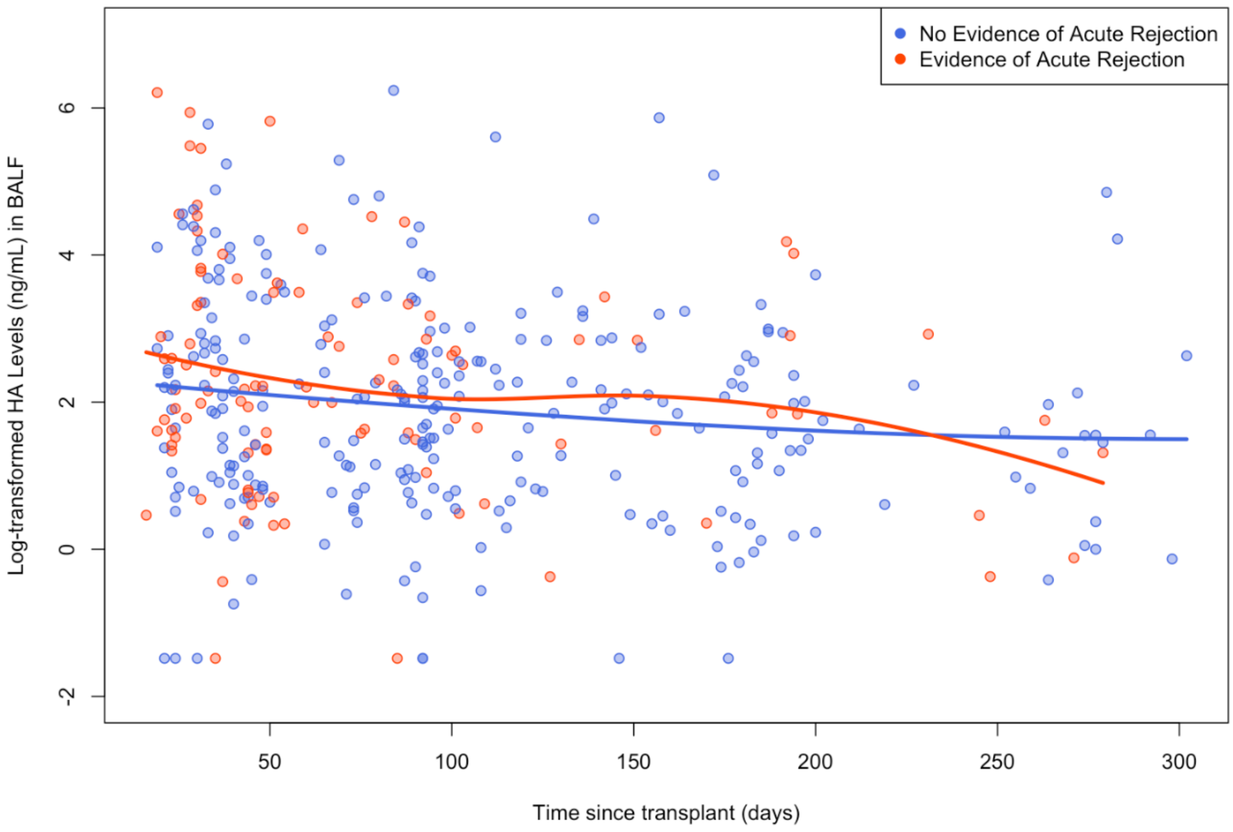

SUPPLEMENTAL TABLES

Table S1. Distribution of acute rejection events and collection times across samples.

		By Acute Rejection (AR) Status	
**Timing of Sample Collection Posttransplant**	**Among BALF Samples (N=373)**	**Samples with Evidence of AR (N=103)**	**Samples with no evidence of AR (N=270)**
< 60 days	144 (38.6%)	59 (57.3%)	85 (31.5%)
61 to 140 days	137 (36.7%)	29 (28.2%)	108 (40.0%)
141 days to 200 days	67 (18.0%)	9 (8.7%)	58 (21.5%)
> 200 days	25 (6.7%)	6 (5.8%)	19 (7.0%)

Table S2. Distribution of patients and samples across the 5 enrolling centers.

Center	Patients N(%)	BAL Samples N(%)
**Cleveland**	52 (41.3%)	162 (43.4%)
**Duke**	44 (34.9%)	143 (38.3%)
**Hopkins**	6 (4.8%)	13 (3.5%)
**Toronto**	5 (4%)	9 (2.4%)
**UCLA**	19 (15.1%)	46 (12.3%)

Table S3. Distribution of infection and nonacute rejection histologies among all samples and as stratified by acute rejection status. The majority of samples had no concurrent infection or nonacute rejection histology.

	**Among All Available BALF Samples (N = 373)**	**Without Acute Rejection (N = 270)**	**With Minimal (A1) Rejection (N = 71)**	**With Mild (A2) Rejection (N = 31)**	**With Moderate (A3) Rejection (N = 1)**
**Infection Status of Paired Biopsy**[*,+]					
No Infection	273 (73.2%)	191 (70.7%)	56 (78.9%)	25 (80.6%)	1 (100.0%)
Bacterial Infection	47 (12.6%)	37 (13.7%)	8 (11.3%)	2 (6.5%)	0 (0.0%)
Viral Infection	34 (9.1%)	27 (10.0%)	5 (7.0%)	2 (6.5%)	0 (0.0%)
Fungal Infection	22 (5.9%)	16 (5.9%)	5 (7.0%)	1 (3.2%)	0 (0.0%)
Mycobacterial Infection	12 (3.2%)	10 (3.7%)	1 (1.4%)	1 (3.2%)	0 (0.0%)
**Concomitant Histology Status of Paired Biopsy**					
No Concomitant Histology	293 (78.6%)	219 (81.1%)	58 (81.7%)	16 (51.6%)	0 (0.0%)
Lymphocytic bronchiolitis	18 (4.8%)	7 (2.6%)	4 (5.6%)	7 (22.6%)	0 (0.0%)
C grade rejection	3 (0.8%)	3 (1.1%)	0 (0.0%)	0 (0.0%)	0 (0.0%)
Acute lung injury	19 (5.1%)	12 (4.4%)	3 (4.2%)	3 (9.7%)	1 (100.0%)
Acute pneumonia	1 (0.3%)	1 (0.4%)	0 (0.0%)	0 (0.0%)	0 (0.0%)
Organizing pneumonia	21 (5.6%)	13 (4.8%)	5 (7.0%)	3 (9.7%)	0 (0.0%)
C4D positive	2 (0.5%)	1 (0.4%)	1 (1.4%)	0 (0.0%)	0 (0.0%)
Suspected AMR	2 (0.5%)	1 (0.4%)	1 (1.4%)	0 (0.0%)	0 (0.0%)
Endothelialitis or arteritis	4 (1.1%)	1 (0.4%)	0 (0.0%)	3 (9.7%)	0 (0.0%)
Capillary inflammation	14 (3.8%)	13 (4.8%)	1 (1.4%)	0 (0.0%)	0 (0.0%)
Evidence of aspiration	4 (1.1%)	3 (1.1%)	0 (0.0%)	1 (3.2%)	0 (0.0%)
Parenchymal fibrosis	4 (1.1%)	4 (1.5%)	0 (0.0%)	0 (0.0%)	0 (0.0%)

Table S4. Distribution of HA concentrations as stratified by BAL instillation volume. Data on instillation volume was missing for 3 samples, these samples were excluded from this descriptive summary.

	**Among All BALF Samples N=370**	**Among Samples from Bilateral Lung Transplants N=274**	**Among Samples from Single LungTransplants N=96**
**Samples with Less than 65 mL Instilled**	122 (33.0%)	100 (36.5%)	22 (22.9%)
HA (ng/mL)[a]	5.6 (1.9, 16.4)	6.5 (2.0, 17.3)	5.1 (1.7, 8.6)
Log-transformed HA (log-ng/mL)[a]	1.7 (0.7, 2.8)	1.9 (0.7, 2.9)	1.6 (0.5, 2.2)
**Samples with 65 to 100 mL Instilled**	171 (46.2%)	122 (44.5%)	49 (51.0%)
HA (ng/mL)	7.3 (2.3, 20.2)	9.2 (2.9, 29.1)	4.7 (1.9, 11.6)
Log-transformed HA (log-ng/mL)	2.0 (0.9, 3.0)	2.2 (1.1, 3.4)	1.6 (0.7, 2.4)
**Greater than 100 mL Instilled**	77 (20.8%)	52 (19.0%)	25 (26.0%)
HA (ng/mL)	8.0 (4.6, 16.3)	8.3 (4.9, 17.9)	8.0 (4.3, 11.5)
Log-transformed HA (log-ng/mL)	2.1 (1.5, 2.8)	2.1 (1.6, 2.9)	2.1 (1.5, 2.4)

[a]HA and Log-transformed HA data are represented as median (1st, 3rd Quartile)

Table S5. Sensitivity analysis examining the effect of enrolling transplant center on the association between BALF HA concentration and biopsy proven acute rejection. Results of the sensitivity analysis are shown side-by-side with those of the primary analysis for comparison.

	**Primary Analysis**				**Sensitivity Analysis**	
	**Unadjusted[a]**		**Adjusted[b]**		**Adjusted[c]**	
	**Effect Estimate (95% CI)**	***P***	**Effect Estimate (95% CI)**	***P***	**Effect Estimate (95% CI)**	***P***
**Acute Rejection (AR)**		0.011		0.044		0.031
Additive Change in Means on the Log-Scale	0.37 (0.09, 0.66)		0.31 (0.01, 0.60)		0.31 (0.03, 0.59)	
Multiplicative Change in Means on the Original-Scale	1.45 (1.09, 1.93)		1.36 (1.01, 1.83)		1.36 (1.03, 1.80)	

[a]Unadjusted model includes the covariate and time of sample collection.

[b]Adjusted model includes AR-status, concomitant infection status, other concomitant histology status, and volume instilled in addition to the covariate and time of sample collection.

[c]Volume instilled removed from adjustment list and replaced with center.

Table S6. Association between %-lymphocyte count in the BALF and BALF HA level.

	Unadjusted[1]		Adjusted[2]	
	**Effect Estimate (95% CI)**	**P-Value**	**Effect Estimate (95% CI)**	**P-Value**
**%-Lymphocyte (log-%)**		0.003		0.004
Additive Change in Means on the Log-Scale	0.18 (0.06, 0.30)		0.18 (0.06, 0.30)	
Multiplicative Change in Means on the Original-Scale	1.20 (1.07, 1.35)		1.19 (1.06, 1.34)	

[1] Unadjusted model includes the covariate and time of sample collection.
[2] Adjusted model includes AR-status, concomitant infection status, other concomitant histology status, and volume instilled in addition to the covariate and time of sample collection.
